# Widespread Hypermetabolism in Symptomatic and Asymptomatic Episodes in Kleine-Levin Syndrome

**DOI:** 10.1371/journal.pone.0093813

**Published:** 2014-04-03

**Authors:** Yves Dauvilliers, Sophie Bayard, Régis Lopez, Frederic Comte, Michel Zanca, Philippe Peigneux

**Affiliations:** 1 Service de Neurologie, Hôpital Gui-de-Chauliac, CHU Montpellier, Montpellier, France; 2 Centre de Référence Nationale Maladie Rare, Narcolepsie et Hypersomnie Idiopathique, Montpellier, France; 3 Inserm U1061, Montpellier, France; 4 Service de Médecine Nucléaire, Hôpital Gui-de-Chauliac, CHU Montpellier, Montpellier, France; 5 UR2NF, Neuropsychology and Functional Neuroimaging Research Unit, Université Libre de Bruxelles, Bruxelles, Belgium; Charité - Universitätsmedizin Berlin, Germany

## Abstract

**Background:**

No reliable biomarkers are identified in KLS. However, few functional neuroimaging studies suggested hypoactivity in thalamic and hypothalamic regions during symptomatic episodes. Here, we investigated relative changes in regional brain metabolism in Kleine-Levin syndrome (KLS) during symptomatic episodes and asymptomatic periods, as compared to healthy controls.

**Methods:**

Four drug-free male patients with typical KLS and 15 healthy controls were included. ^18^-F-fluorodeoxy glucose positron emission tomography (PET) was obtained in baseline condition in all participants, and during symptomatic episodes in KLS patients. All participants were asked to remain fully awake during the whole PET procedure.

**Results:**

Between state-comparisons in KLS disclosed higher metabolism in paracentral, precentral, and postcentral areas, supplementary motor area, medial frontal gyrus, thalamus and putamen during symptomatic episodes, and decreased metabolism in occipital and temporal gyri. As compared to healthy control subjects, KLS patients in the asymptomatic phase consistently exhibited significant hypermetabolism in a wide cortical network including frontal and temporal cortices, posterior cingulate and precuneus, with no detected hypometabolism. In symptomatic KLS episodes, hypermetabolism was additionally found in orbital frontal and supplementary motor areas, insula and inferior parietal areas, and right caudate nucleus, and hypometabolism in the middle occipital gyrus and inferior parietal areas.

**Conclusion:**

Our results demonstrated significant hypermetabolism and few hypometabolism in specific but widespread brain regions in drug-free KLS patients at baseline and during symptomatic episodes, highlighting the behavioral state-dependent nature of changes in regional brain activity in KLS.

## Introduction

Kleine-Levin syndrome (KLS) is an extremely rare disease characterized by recurrent episodes of hypersomnia associated with at least one of the following symptoms: cognitive abnormalities, behavioral disturbances, hyperphagia or hypersexuality [1]–[3]. Nowadays diagnostic criteria for KLS are only clinically defined [3]. No reliable biomarker has been identified yet, and the underlying pathophysiology of KLS remains unknown. So far, four post-mortem brain examinations have been reported with inconsistent results [4], [5]. However, inflammatory encephalitis within thalamic and hypothalamic regions was found in two patients. Results of the few functional neuroimaging studies conducted in KLS during and between symptomatic episodes are also conflicting regarding changes in brain activity, particularly in thalamic, hypothalamic, basal ganglia structures and in fronto-temporal areas [6]–[12]. Inconsistencies may stem from the use of different imaging methods (single photon emission tomography [SPECT], ^18^F-fluorodeoxyglucose positron emission tomography [^18^F-FDG-PET]), small sample size, imaging at different stages of disease evolution and delay from episode onset, various phenotypes during imaging recording, and medication intake. Moreover, to the best of our knowledge no PET-scan imaging studies compared patients with KLS to healthy controls.

In the present study, brain regional metabolism was investigated using ^18^F-FDG-PET scan in 4 typical boys with KLS during both symptomatic and asymptomatic states, and data were compared to 15 healthy controls.

## Methods

### Participants

All subjects and both parents on behalf of the minors enrolled in the study gave their written informed consent to participate in, study being approved by the Montpellier University Hospital’s ethics committee.

Four male patients were diagnosed with typical KLS [3], with onset of symptoms at 15–16 years, 2 to 10 episodes of hypersomnia and derealisation/behavioral disturbances since the beginning with a duration ranging from 4 to 10 days (Table 1). Two patients presented disinhibition, hypersexuality and increased food intake without megaphagia. All patients experienced derealization during symptomatic episodes. Each patient was hospitalized twice for 2 days during symptomatic and asymptomatic episodes to record sleep, performed clinical and neuropsychological examns and underwent ^18^F-FDG-PET scanning at rest (Table 1).

**Table 1:** Demographic, clinical and neuropsychological characteristics of 4 patients with Kleine-Levin Syndrome.

	Patient 1	Patient 2	Patient 3	Patient 4
**Demographical data**				
Age, years	16	16	17	16
Onset, years	15	15	16	15
Body mass index, kg/m2	22.4	24.2	20.5	23
**Clinical data**				
Time elapsed since the first attack, month	16	11	9	13
Number of attacks since disease onset	10	8	5	2
Duration of a single attack, days	5 to 7	5 to 7	4 to 5	10
First attack precipitating factor	Infection	Alcohol intake	Sleep deprivation	Infection
**Questionnaires**				
Epworth Sleepiness Scale score*	17_5	16_5	15_4	16_3
Fatigue severity scale*	9_2	8_1	6_0	9_0
**Sleep abnormalities**				
Hypersomnia	+	+	+	+
Sleep inertia	+	+	+	+
Postepisode transient insomnia	−	−	−	−
Intense dreaming	−	−	−	−
Hypnagogic hallucinations	−	−	−	−
Sleep paralysis	−	−	−	−
**Sexual disinhibition**				
Disinhibition, hypersexuality	+	−	−	+
Increased masturbation	+	−	−	+
**Eating behaviour**				
Compulsive eating	−	−	−	−
Increased food intake	+	−	−	+
Decreased of appetite	−	+	+	−
**Altered perception**				
Erroneous perception	−	+	−	−
Derealization	+	+	+	+
**Psychological changes**				
Irritability	+	−	+	+
Depressed mood	−	−	−	−
Agitation	−	−	−	−
Less polite	+	−	−	+
Anxiety	−	−	−	−
Compulsions	−	−	−	−
**PET-scan**				
in asymptomatic episode	one month after 9th	one month after 7th	one month after 5th	3 months after 2nd
in symptomatic episode	10th attack, day 3	8th attack, Day 2	5th attack, Day 2	2nd attack, Day 3

* = during last attack_ in between attack; − = Absent;+ = Present.

All participants were drug-free at the time of evaluation; none was previously treated for KLS. Follow-up and clinical re-evaluations of these patients confirmed the accuracy of KLS diagnosis without any associated psychiatric comorbidities, without any residual symptoms in between symptomatic episodes, and suggested disease recovery in patients 1 and 3.

Fifteen healthy controls (12 males, mean age 28±14.9, range 10–51 years) participated in the study that included 10 young subjects with a mean age 17.9 years. Healthy controls were identified with normal PET scans, All controls had normal neurological exams, normal and regular sleep, were free of any treatment that may interfere with sleep, motor or psychological functions, None of the controls suffered from major depression or any psychiatric disorder as assessed by clinical interview.

### PET Procedure

During symptomatic episodes, PET scans were all performed Day 2 to Day 3 after episode onset as detailed in table 1. PET scans in asymptomatic periods were obtained one to three months after the last day of the previous symptomatic episode (Table 1).

All participants received via intravenous line an injection of 5.5 MBq/kg of FDG, in a quiet room between 10∶00 a.m. and 1∶00 p.m., with eyes closed to avoid noxious, auditory or visual stimuli. Before the injection and up to PET scan acquisition, all participants were asked ‘to resist sleeping’ in order to remain fully awake during the procedure, but also not to move and not to talk. The vigilance state of patients and controls was supervised clinically and by video by both a neurologist (YD) and a technician during the whole process. None of the subjects slept during the PET scan acquisition.

PET images were acquired during 15 min using a Biograph Emission Duo LSO (Siemens Corp) camera 30 minutes after FDG injection. The volume was reconstructed using a FORE rebinning algorithm followed by an OSEM reconstruction with 16 subsets and eight iterations, following the manufacturer’s operating instructions. The resulting images contained 47 contiguous slices with a plane separation of 5 mm.

### Data Analysis

Metabolic data were analysed using SPM8 software (Wellcome Department of Cognitive Neurology, Institute of Neurology, London, UK) implemented in MATLAB (Mathworks, Sherborn, MA). Images were spatially normalized into the ICBM standard space using 2^nd^ degree B-spline interpolations, then smoothed using a 16-mm full-width half-maximum isotropic Gaussian kernel.

Areas of significant relative (instead of absolute) change between conditions of interest were estimated according to the general linear model using linear contrasts. Global metabolism adjustment was performed using proportional scaling. Age was entered in the analysis as a confounding variable. Main contrasts estimated the main effect of pathology [controls vs. KLS at baseline, controls vs. KLS in acute episode] to identify the brain regions where glucose metabolism was decreased (or increased) in the KLS as compared to controls. In a second step, the condition effects were assessed within the KLS population [baseline vs. acute episode] to identify brain areas where glucose metabolism was decreased (or increased) as a function of the presence of acute symptoms in KLS. The resulting set of voxel values for each contrast constituted a map of the T statistics [SPM{T}], thresholded at p ≤.001 (T≥3.35), uncorrected (comparisons with controls) or p ≤.05 (T≥1.71), uncorrected (direct comparison between acute and baseline state in KLS). Minimal spatial extent of reported brain areas is 50 contiguous significant voxels.

## Results

### Comparison of Cerebral Glucose Metabolism between Symptomatic and Asymptomatic Episodes in KLS

**Figure 1:**
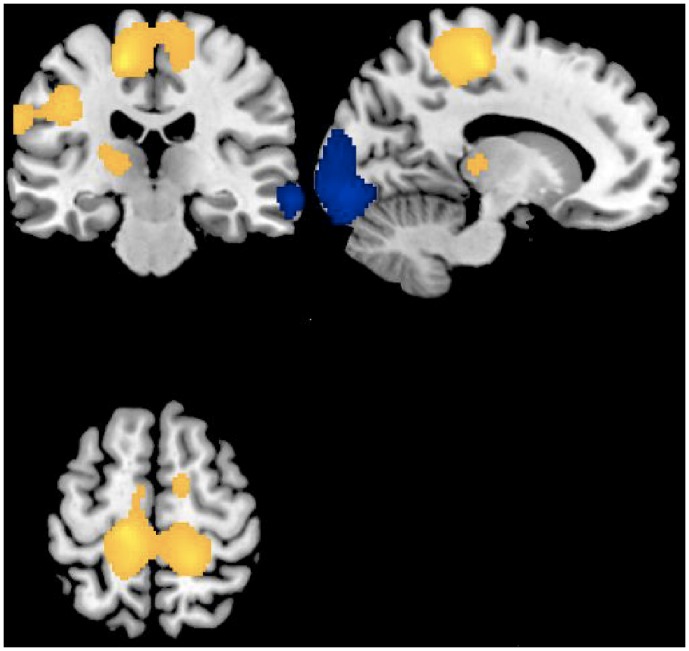
Regional cerebral increases (yellow blobs) and decreases (blue blobs) in Kleine-Levin patients (n = 4) during symptomatic episodes as compared to baseline asymptomatic period, superimposed on a template T1-weighted MRI. Activations are displayed at p<.05, uncorrected, for clusters >50 voxels.

A direct comparison between states in KLS patients showed a higher relative metabolism during the symptomatic episodes in paracentral, precentral, and postcentral areas, supplementary motor area, superior medial frontal gyrus and in the left thalamus and putamen (lentiform nucleus) bilaterally (Table 2, Fig. 1). Noticeably, a statistical trend for hypermetabolism was similarly found within the thalamus and putamen during symptomatic KLS episodes compared to healthy controls, without any changes within these areas between KLS at baseline and controls. Conversely, relative lower metabolism was found during symptomatic KLS episodes bilaterally in occipital gyri, in left lingual gyrus and right angular and middle and superior temporal gyri (Table 2, Fig. 1).

**Table 2:** Statistical parametric mapping results of brain regions showing relative metabolism changes during symptomatic episodes and asymptomatic periods in Kleine-Levin Syndrome KLS (n = 4).

	Hemisphere	Coordinates			
A. Episodes vs. Baseline	(L)eft/(R)ight	x	y	z	Z-score
Precentral gyrus	L	−16	−24	56	3.23
Postcenral gyrus	R	16	−38	64	2.74
Supplementary motor area	R	14	−4	54	2.27
Postcenral gyrus	L	−64	−14	26	2.76
Paracentral lobule	R	8	−14	44	1.86
Superior Medial Gyrus		0	68	28	2.21
Thalamus	L	−18	−26	6	1.78
Putamen (lentiform nucl.)	R	28	−18	14	1.87
Putamen (lentiform nucl.)	L	−32	−12	6	1.78
**b. Baseline vs Episodes**	**(L)eft/(R)ight**	**x**	**y**	**z**	**Z-score**
Inferior Occipital Gyrus	R	28	−94	−2	3.29
Lingual Gyrus	L	−28	−96	−16	2.79
Middle Occipital Gyrus	L	−24	−92	2	2.82
Angular Gyrus	R	38	−74	32	2.29
Middle Temporal Gyrus	R	68	−30	−10	2.78
Superior Temporal Gyrus	R	62	−54	14	2.37

Note. Coordinates x, y, z refer to the standard Talairach and Tournoux^33^ stereotactic space. Activation peaks’ significance is reported at the voxel-level, thresholded at p ≤.05, with a cluster extent >50 voxels. Only the most representative voxels in each anatomical structure are displayed.

Inspection of individual data showed that increased (respectively decreased) regional glucose consumption was consistent across the 4 KLS patients who exhibited similar differential patterns.

### Regional Cerebral Glucose Metabolism in KLS at Baseline Compared to Healthy Controls

**Figure 2:**
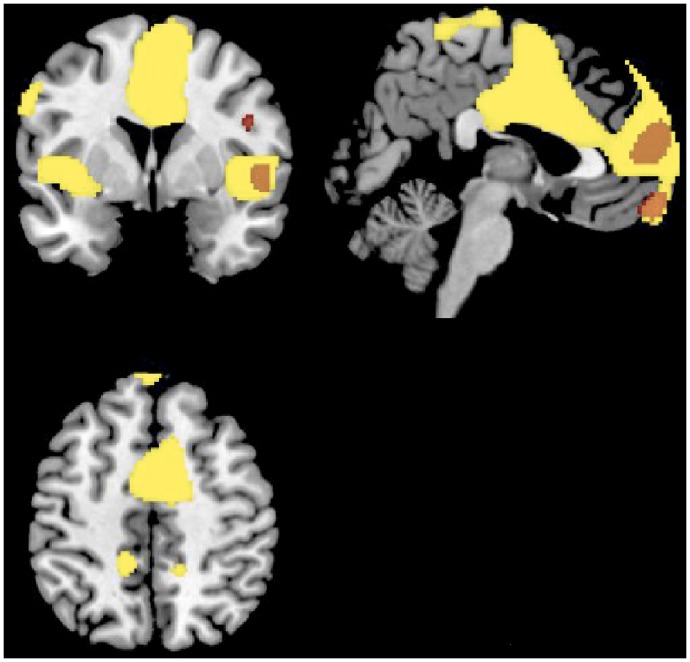
Regional cerebral increases in Kleine-Levin patients as compared to controls during symptomatic episodes (yellow) and asymptomatic period (red), superimposed on a template T1-weighted MRI. Activations are displayed at p<.001, uncorrected, for clusters >50 voxels.

A relative increased brain regional metabolism was found in KLS patients at baseline (i.e. in asymptomatic periods) compared to healthy controls in the medial, middle and inferior frontal cortices, inferior and middle temporal gyri, left posterior cingulate, and the right precuneus (Table 3; Fig. 2). Conversely, no hypometabolism was found.

**Table 3:** Statistical parametric mapping results of brain regions showing relative metabolism changes in patients with Kleine-Levin Syndrome KLS (n = 4, baseline) compared to healthy controls (n = 15).

	Hemisphere	Coordinates			
Hypermetabolism in KLS	(L)eft/(R)ight	x	y	z	Z-score
Medial Frontal Gyrus	L	−6	60	−16	4.47
Medial Frontal Gyrus	R	12	64	12	4.46
Inferior frontal gyrus	R	48	4	24	3.67
Middle frontal gyrus	R	36	40	12	3.63
Middle frontal gyrus	R	60	30	28	3.63
Inferior Temporal Gyrus	R	52	−28	−22	4.17
Middle Temporal Gyrus	R	66	−24	−12	4.26
Superior Temporal Gyrus	R	54	6	2	3.46
Precuneus	R	24	−54	22	4.07
Posterior cingulate	L	−18	−56	18	3.49

Note. Coordinates x, y, z refer to the standard Talairach and Tournoux^33^ stereotactic space. Activation peaks’ significance is reported at the voxel-level, thresholded at p ≤.001, with a cluster extent >50 voxels. Only the most representative voxels in each anatomical structure are displayed.

### Regional Cerebral Glucose Metabolism during KLS Episodes Compared to Healthy Controls

A relative increased brain metabolism was found during symptomatic episodes in KLS patients as compared to healthy controls in inferior, superior medial and orbital frontal areas, supplementary motor area, left lingual and right inferior and superior temporal gyri, precuneus, precentral and posterior regions, left insula and inferior parietal areas, and right caudate nucleus in the striatum. Hypometabolism was found in the middle occipital gyrus bilaterally, in right inferior parietal areas and in the left cuneus (Table 4, Fig. 2).

**Table 4:** Statistical parametric mapping results of brain regions showing relative metabolism changes during symptomatic episodes in patients with Kleine-Levin Syndrome KLS (n = 4, acute episode) compared to healthy controls (n = 15).

	Hemisphere	Coordinates			
a. Hypermetabolism in KLS	(L)eft/(R)ight	x	y	z	Z-score
Inferior frontal gyrus	L	−42	12	22	3.54
Superior Medial Frontal	R	12	64	12	5.13
Superior Orbital Frontal	L	−18	18	−16	3.58
Supplementary motor area	R	12	0	54	4.62
Left Insula	L	−38	−2	4	3.93
Right Caudate Nucleus	R	12	14	−12	3 47
Inferior Temporal Gyrus	R	50	−26	−22	4.25
Superior Temporal Gyrus	R	54	4	2	3.79
Inferior Parietal Lobule	L	−48	−54	38	3.76
Postcentral Gyrus	L	−54	−2	42	3.52
Precentral Gyrus	L	−42	−8	40	3.61
Precentral Gyrus	L	−60	2	34	3.47
Precuneus	R	22	−52	22	4.29
Precuneus	L	−16	−54	16	3.96
Lingual gyrus	L	−18	−42	−2	3.28
**b. Hypometabolism in KLS**					
Middle Occipital Gyrus	R	28	−96	8	4.40
Middle Occipital Gyrus	L	−22	−100	12	3.61
Angular Gyrus	R	38	−74	32	4.02
Inferior Parietal Gyrus	R	66	−44	24	3.63
Cuneus	L	−14	−104	−4	3.51

Note. Coordinates x, y, z refer to the standard Talairach and Tournoux^33^ stereotactic space. Activation peaks’ significance is reported at the voxel-level, thresholded at p ≤.001, with a cluster extent >50 voxels. Only the most representative voxels in each anatomical structure are displayed.

To minimize the confounding factor of age, similar analyses were conducted comparing FDG-PET patterns with young control participants (n = 10; mean age 17.9 years). These analyses disclosed similar results although effects were less pronounced in cortical areas, especially in frontal cortices, which is likely due to the loss of statistical power.

## Discussion

We report here the results of a case-control ^18^F-FDG-PET scan study in KLS during and between episodes. Between states comparison within KLS consistently revealed higher metabolism during symptomatic episodes in paracentral, precentral, postcentral and supplementary motor areas, frontal gyrus, thalamus and putamen, and hypometabolism in the occipital and temporal gyri. We also demonstrated significant.

hypermetabolism and few hypometabolism in specific but widespread cortical network in drug-free fully awake KLS patients at baseline and during symptomatic episodes compared to healthy controls.

Our results may contrast from previous SPECT studies having suggested hypoperfusion particularly within the thalamus but also in the temporal and frontal lobes and basal ganglia during symptomatic episodes [6]–[10]. However, no control group was included and no statistical parametric mapping analysis of SPECT images was available. A KLS case report using FDG PET-scan showed decreased metabolism in the hypothalamus, caudate nuclei and striatum during symptomatic as compared to asymptomatic phases but the quantitative method was a simple subtraction without statistical evaluation of the observed differences [11]. More recently, a PET-scan study conducted on two KLS cases reported more complex regional metabolism modifications with decreased glucose consumption in the hypothalamus, frontal areas and posterior regions but also increased activity in anterior caudate, cingulate and the premotor cortex during symptomatic episodes [12].

Discrepancies and variability in metabolic patterns between KLS studies [4], [5] and with our results may stem from various factors including the image scanning method (e.g. SPECT vs. PET), the type of analysis (mere subtraction, statistics in the general linear model), time of imaging after the onset of the symptomatic episodes (i.e. here, PET scans were all performed Day 2 to Day 3 after episode onset), participants’ age and disease evolution, medication intake (i.e. lithium) [12], the limited number of patients included, and in particular the variable phenotype during actual imaging recording (awake vs. sleepy, presence of derealization, hypersexuality, hyperphagia…). The latter issue is of major concern since disease symptoms are not stable between episodes for a single patient, nor between patients [1], [2], [4], [13]. Post-mortem brain examination also revealed large between-patient differences regarding the presence of inflammation and lesion localizations [4], [5]. However, inspection of individual images disclosed consistent pattern of metabolism changes in the main areas across our four participants, a consistency potentially related to an homogeneous age group being awake and drug-free at the time of scanning.

We reported here a systematic and controlled comparison between KLS at baseline and during symptomatic episodes and healthy controls. Although most of literature findings suggested potential temporal, frontal and basal ganglia hypoperfusions persisting during asymptomatic periods after many recurrences and years of disease duration, these conclusions are hampered by a lack of comparisons with healthy controls [2], [4], [5], [6]–[12]. Our results disclose restricted regional hypometabolism during symptomatic episodes, and none in the asymptomatic periods even though our patients were young, with recent disease onset and in disease regression for two of them. In contrast, we found increased brain metabolism in a specific but widespread set of brain regions in KLS in asymptomatic periods but especially during symptomatic episodes compared to controls even in the subanalysis including the young control participants only. Regional hypometabolism in occipital, lingual and temporal gyri during symptomatic episodes may play a role in the pathophysiology of delusions, derealization, and disinhibition [4], [5], [13]. Conversely, hypermetabolism within the supplementary motor area, frontal gyri, thalamus and putamen may explain some behavioral symptoms, may reflect a compensatory activity for hypoactivated adjacent brain areas [4], [5], [13], but may also reflect the vigilance state during symptomatic episodes. Indeed, sleep-deprivation studies in normal controls have reported that poor performance elicit an attentional recovery that may manifests as greater activation in the prefrontal cortex, parietal lobe and cingulate regions [14]. This observation might be related to the increased activation patterns found in KLS patients during a working memory task, reflecting higher efforts than controls [15]. We previously reported hypermetabolism in drug-free patients with narcolepsy-cataplexy in fully awake condition in the cingulated cortex, cuneus and lingual gyrus compared to controls [16]. Thus, we suggest that the vigilance level during the scanning procedure is a critical issue in central hypersomnias. Investigation of brain functional imaging in patients with central hypersomnia should be performed in well-defined vigilance state, i.e. in wakefulness state to avoid results showing altered functional neurocircuitry secondary to sleepiness. None of our subjects slept during the scanning acquisition as controlled clinically and by video; however unfortunately the vigilance state was not monitored through EEG recording during the PET procedure.

In conclusion, we demonstrated significant hypermetabolism and few hypometabolism in specific but widespread brain regions in drug-free fully awake KLS patients at baseline and during symptomatic episodes compared to healthy controls, highlighting the behavioral state-dependent nature of global cortical function. Further studies are needed in larger populations to elucidate the functional activation/inhibition of brain regions involved in particular symptoms of KLS patients.
